# State Board of Health—Annual Examinations

**Published:** 1882-05

**Authors:** 


### ARTICLE XV.

ILLINOIS STATE BOARD OF HEALTH,
SPRINGFIELD, Illinois, April 17, 1882.

MESSRS. EDITORS: Accompanying this, please find the schedules of questions used at the last annual examination of candidates for certificates of practice by the Illinois State Board of Health, (Chicago, April 13–15, 1882), and to which questions 80 per cent. of correct answers are required.

The numerous inquiries, both from within and without the State, as to the scope and character of these examinations indicate that the publication of the questions may be timely and seful.

It should be noted that, after the session of 1882–83, candidates will be also required to undergo an examination in the branches of a good English education, including mathematics, English composition and elementary physics or natural philosophy. Very respectfully,

JOHN H. RAUCH, M. D., Secretary.

*To the Editor Chicago Medical Journal and Examiner.*

ILLINOIS STATE BOARD OF HEALTH, Chicago, April 13, 1882.

*Examination in Anatomy—By W. A. Haskell, M. D.* 1. Name the bones of the carpus. 2. With what bones does the sphenoid articulate? 3. Describe a vertebra. 4. Describe the ligaments of the hip joint. 5. Name and describe the pronator muscles of the forearm. 6. Give the relations of the femoral artery and vein. 7. Describe the thoracic duct. 8. Give the distribution of the median nerve. 9. What is Wharton's duct? 10. Describe the liver.

*Examination in Surgery—By W. A. Haskell, M. D.* 1. Define inflammation. 2. What is the difference between ulceration and mortification? Between caries and necrosis? 3. What is a tumor? 4. Give illustrations of a benign and of a malignant tumor. 5. Give the treatment of mammary abscess? 6. Explain the *modus operandi* of reduction of the iliac dislocation of the head of the femur, by manipulation. 7. Give the differential diagnosis of compression and concussion of the brain. 8. Give the differential diagnosis of inguinal hernia and hydrocele of the cord. 9. Give the diagnosis of morbus coxarius—(a) During the first stage before the occurrence of effusion. (b) During the stage of effusion—the capsule of the joint remaining entire. 10. Give the general treatment of fractures of the lower extremities.

*Examination in Physiology—By John McLean, M. D.* 1. What is the action of saliva in digestion, and what are its chemical constituents? 2. Describe the digestion of starch and of fats. 3. Give the source and use of animal heat. 4. How is gastric juice formed, and what is its composition? 5. Explain the secretion of bile, its composition and use. 6. Explain the physiology of sleep. 7. Describe the fœtal circulation. 8. What nerves are directly concerned in the act of respiration?

9. Describe the circulation of blood in the fœtal heart. 10. What causes the sounds of the heart?

*Examination in the Practice of Medicine—By John McLean, M. D.* 1. What are the symptoms of variola, and its treatment? 2. How would you diagnose variola from varicella? 3. Give etiology, pathology and treatment of cholera infantum. 4. What is hysteria, and its treatment? 5. Give etiology, pathology and treatment of epilepsy. 6. Give diagnosis and treatment of eczema squamosa. 7. Give pathology, causes and treatment of typho-malarial fever. 8. Give differential diagnosis of diphtheria, and its treatment. 9. Give symptoms and treatment of leucocythæmia. 10. Give symptoms and treatment of acute idiopathic erysipelas.

*Examination in Materia Medica and Therapeutics—By J. H. Rauch, M. D.* 1. Classify remedial agents, broadly, by their actions and uses. 2. Name some of the principal agents in each class. 3. Name the principal urino-genital remedies, and write five prescriptions, embracing a different one in each. Give the indications intended to be met by each prescription. 4. What alteratives, emetics and cathartics are indigenous in Illinois? 5. Give the sources, active principles, two or more officinal preparations, and uses of (a) camphor; (b) ergot; (c) nux vomica; (d) opium; (e) physostigma. 6. Describe the therapeutic uses of the bromides, and write prescriptions for each of three of them, with indications. 7. Mention some of the most important recent additions to the materia medica, with their uses. 8. Give the therapeutic uses and applications of *aqua fluvialis* or *fontana.* 9. Mention the different officinal preparations of antimony. 10. Give the doses of (a) ammonii phosphas; (b) iodoformum; (c) strychniæ sulphas; (d) acidum boracicum; (e) extr. belladonnæ alc.; (f) atropiæ sulphas; (g) resina podophylli; (h) tr. aconita rad.; (i) extr. gelsemii fld.; (k) acidum hydrocyanicum dilutum.

*Examination in Hygiene—By J. H. Rauch, M. D.* 1. Give the prophylaxis of small-pox, and the measures to prevent its spread on the appearance of the first case. 2. To what extent should vaccination be made compulsory in the United States, and why? 3. What is "ground-water," and what is its agency on health? 4. Describe the principal disinfectants, their applications and modes of use. 5. Formulate a set of rules for school hygiene. 6. What is "sewer gas," and what evils are ascribed to it? 7. Give the differential diagnosis, for sanitary purposes, of (a) scarlatina; (b) rubeola; (c) varicella; (d) variola; (e) febris flava; (f) cholera Asiatica; (g) trichiniasis. 8. Describe vaccination and its progress through the different stages; the effects ascribed to it; its complications; and the ages at, or conditions under, which it should be repeated. 9. What are the chief causes of an excessive mortality, and their remedies? 10. Describe Pasteur's recent experiments.

*Examination in Medical Jurisprudence—By J. H. Rauch, M. D.* 1. At what age is the fœtus viable, and what are the signs and indications of such age? 2. What precautions—other than for the safety of the subject—would you observe in the exhibition of an anæsthetic, and why? 3. How would you determine whether lesions, injuries or discolorations, found on a cadaver, were produced before or after death? 4. What is the course of procedure in the commitment of persons to an insane asylum in this State? 5. Has the registration of vital statistics any legal bearing, and, if so, what?

*Examination in General Pathology—By R. Ludlam, M. D.* 1. Give a definition of disease. 2. What is the difference between a predisposing and an exciting cause of disease? 3. Name the means employed in physical diagnosis. 4. What is meant by "a qualified prognosis?" 5. What forms of inflammation are reparative? 6. How would you recognize the cancerous cachexia? 7. What diseases are incident to the hæmorrhagic diathesis? 8. In what diseases do we often find albumen in the urine? 9. What form of erysipelas is inoculable? 10. Why do attacks of pelvic and portal congestion frequently alternate wlth each other?

*Examination in Gynæcology—By R. Ludlam, M. D.* 1. What are the uses of the uterine sound? 2. What diseases are accompanied by an increased depth of the womb? 3. In constipation, with or without hæmorrhoids, which ovary is most frequently inflamed, and why? 4. What intra-pelvic inflammation is most frequently rheumatic? 5. What diseases are followed by fixity, or anchorage of the uterus? 6. Name the most frequent cases of menorrhagia in women who have had one or more children. 7. Define a menstrual headache, and give the treatment for it. 8. What are the sources of puerperal traumatism, and what are the most serious lesions that may result from it? 9. In a lying-in patient, how would you distinguish a physiological from a pathological chill? 10. When are mammary abscesses salutary?

*Examination in Obstetrics—By A. L. Clark, M. D.* 1. Define obstetrics. 2. How can you differentiate pregnancy from ovarian tumor or cyst? 3. At what period or stage of labor is the greatest danger to the mother, and what is the danger? 4. Give the contra-indications to the use of ergot. 5. Under what circumstances should version be performed? 6. Will the mother's blood pass out from the umbilical cord unless this be tied before being cut? 7. Give diagnosis and treatment of puerperal eclampsia. 8. Give diagnosis and treatment of hydrocephalus of the infant during parturition. 9. What is the shape of the posterior fontanel? 10. Give the treatment for prolapse of the funis umbilicalis.

*Examination in Chemistry—By A. L. Clark, M. D.* 1. What is meant by a qualitative, and what by a quantitative, analysis? 2. How would you test water for organic impurities? 3. Is hard or soft water most liable to contamination by passage through or standing in lead pipes, and why? 4. How would you test a suspected water for salts of lead in solution? 5. Give the names and symbols for ten elementary substances? 6. Name substances with which it is incompatible to unite KI in prescriptions. 7. What chemical elements are contained in pure grape sugar not found in cane sugar? 8. What liquid is the most universal solvent? 9. What is the difference between analysis and synthesis? 10. What precautions are necessary in handling chloroform in the presence of flame or fire?

